# Role of ultrasound, clinical and scintigraphyc parameters to predict malignancy in thyroid nodule

**DOI:** 10.1186/1758-3284-3-17

**Published:** 2011-03-22

**Authors:** Frederico FR Maia, Patrícia S Matos, Bradley P Silva, Ana T Pallone, Elizabeth J Pavin, José Vassallo, Denise E Zantut-Wittmann

**Affiliations:** 1 Endocrinology Division, Department of Internal Medicine, University of Campinas, São Paulo, Brazil; 2 Department of Pathology, Medical Science School, University of Campinas, São Paulo, Brazil

## Abstract

**Background:**

This study aimed to evaluate clinical, laboratory, ultrasound (US) and scintigraphyc parameters in thyroid nodule and to develop an auxiliary model for clinical application in the diagnosis of malignancy.

**Methods:**

We assessed 143 patients who were surgically treated at a single center, 65% (93) benign vs. 35% (50) malignant lesions at final histology (1998-2008). The clinical, laboratory, scintigraphyc and US features were compared and a prediction model was designed after the multivariate analysis.

**Results:**

There were no differences in gender, serum TSH and FT4 levels, thyroid auto-antibodies (TAb), thyroid dysfunction and scintigraphyc results (P = 0.33) between benign and malignant nodule groups. The sonographic study showed differences when the presence of suspected characteristics was found in the nodules of the malignant lesions group, such as: microcalcifications, central flow, border irregularity and hypoechogenicity. After the multivariate analysis the model obtained showed age (>39 years), border irregularity, microcalcifications and nodule size over 2 cm as predictive factors of malignancy, featuring 81.7% of accuracy.

**Conclusions:**

This study confirmed a significant increase of risk for malignancy in patients of over 39 years and with suspicious features at US.

## Introduction

Thyroid nodule is a common clinical problem. Epidemiologic studies have shown that the prevalence of palpable thyroid nodules are found in approximately 5% of women and in 1% of men living in iodine-sufficient parts of the world [1,2]. On the other hand, ultrasound (US) studies could detect thyroid nodules in 19-67% of selected individuals with higher frequencies mainly in women and elderly people [3]. The majority of patients with thyroid nodule can be managed conservatively and it justifies the effort to select better candidates for thyroidectomy [4-6].

A number of clinical, US, and cytological parameters have been previously studied; however, none of them have shown significant impact on clinical practice [6]. Molecular markers are promising but they have not yet been sufficiently validated to be used in clinical practice [7,8]. The role of clinical evaluation of patients who have thyroid nodule is to minimize the risk of overlooking thyroid cancer.

When clinical, laboratory and US parameters are employed, there is an increase of suspicion for malignancy. It includes age (< 20 or > 70 yrs.), gender (male), large size (> 4 cm or > 2 cm in recent series), serum thyrotropin (TSH) levels (even in normal ranges: > 1.8 mU/ml), positive thyroid auto-antibodies (TAb) and scintigraphyc study of cold nodules [9-12]. In addition, it has been widely perceived that malignancy rates are higher in subjects with solitary nodules than in those affected with multinodular goiters [5,7,9]. Although, recent data showed that there is no correlation among TSH levels, thyroid autoimmunity and central nodule flow on US and color Doppler scans of thyroid cancer [13-15]. A current study proposed a risk score analysis based on patient's age (50 years), nodule size (2.5 cm) and cytopathological features (atypia) in patients who presented indeterminate or suspicious fine-needle aspiration (FNA) [16].

The accuracy of these clinical and laboratory aspects, US or scintigraphic features in distinguishing benign from malignant nodules is not well established [17,18]. This study aimed to verify predictive factors in clinical, laboratory, US and at scintigraphyc tests, which suggest malignancy in thyroid nodules, and to develop an auxiliary diagnosis model in clinical applications for management of thyroid nodules.

## Materials and methods

### Population Study - Clinical Parameters

We retrospectively studied the data from 151 patients with 194 nodules who were submitted to total or partial thyroid surgery between 1998 and 2008 at a General Hospital of University of Campinas, Brazil. All patients preoperatively diagnosed with a thyroid nodule by US or clinical examination underwent ultrasound-guided fine-needle aspiration cytology (US-FNAC), and were assessed retrospectively for clinical, laboratory, US and scintigraphyc variables. From the total sample 51 thyroid lesions and eight patients were excluded because they lacked enough information and criteria for statistical analysis. This study included 65% (93) benign vs. 35% (50) malignant lesions at final histology result and a follow up of patients for 33.9 ± 41.7 months. Surgery decision was made based on clinical (laboratory and US features), cytological and image criteria (compressive symptoms) for all cases. Indeterminate cytology was the most common surgical indication (Table 1).

**Table 1:** Criteria for Surgical Treatment of Thyroid Nodule in a Single Center

Surgical Criteria	N	%
Clinical/Compressive symptoms	26	18.7%
Inconclusive FNAC	14	10.2%
Scintigraphy - Cold Nodule	06	4.3%
FNAC diagnosis	13	9.35%
FNAC suspect (indeterminate)	50	35.9%
FNAC suspect + US features	27	19.4%
US suspect features	03	2.15%

FNAC: fine-needle aspiration cytology; US: ultrasound.

Clinical variables included age and gender, and the demographic information took into account the patient's age (≥ 45 yrs.). Women were predominant in the two groups (benign vs. malignant nodules) (Table 2).

**Table 2:** Clinical and Laboratory Variables of Patients under Thyroid Nodule Evaluation in a Single Center

Variables	Benign	Malignant	Total	*P-*value
Patients (n)	93	50	143	-
Age (months)	48.6 ± 11.9	44.6 ± 16.5	47.2 ± 13.7	0.23
Age ≥ 45 yrs. (%)	68.5%	31.5%	65%	0.24
Gender (Female) (%)	86%	51.3%	84.6%	0.52
TSH (mU/ml) [median]	1.37	1.82	1.47	0.11
FT4 (mU/ml) [median]	1.23	1.28	1.24	0.51
TAb positively (%)	23.6%	15.6%	21.2%	0.36
Auto-Immune Disease (%)	24.7%	14.7%	21.5%	0.24
Normal Thyroid function (n)	68	42	110	0.30
Hyperthyroidism (Graves) (n)	15	04	19	0.61
Hypothyroidism(n)	10	04	14	0.15

Laboratorial variables involved TSH and free thyroxin (FT4) levels as the baseline. TSH and FT4 were dosed using a chemiluminescence's analyzer, and a sandwich technique on Roche Elecsys immunoassay analyzer, which ranged from 0.4 to 4.5 UI/ml, and had intra-assay variation: 13.8%; inter-assay variation: 17.5% for the TSH and 0.9 to 1.8 ng/ml; intra-assay variation: 6.8%; inter-assay variation: 7.8% for FT4. Thyroid autoimmunity was defined considering elevated levels of antithyperoxidase antibody (TPO-Ab), determined by immunometric assays (reference value < 35 μUI/ml), intra-assay variation: 4.3%, inter-assay variation: 10.5% and antithyroglobulin antibody (Tg-Ab, reference value < 49 IU/ml); intra-assay variation: 2.3%; inter-assay variation: 8.1%.

We classified thyroid disorders in normal thyroid function (TSH and FT4 values within the reference ranges) as follows: autoimmune thyroid disease (AITD) (euthyroid with elevated TAb); overt hypothyroidism, elevated TSH with reduced free T4 levels); subclinical hypothyroidism (elevated TSH and normal FT4); thyrotoxicosis (low TSH and elevated FT4) and subclinical hyperthyroidism (low TSH and normal free T4 and T3).

### Scintigraphyc Features

The relevance of the cold nodule was evaluated using a ^99 m^Tc-pertechnetate (Tc) scan. Twenty minutes after intravenous injection of 10 mCi (370 MBq), ^99 m^Tc images were obtained on a computerized scintillation camera equipped with a low-energy, high-resolution, parallel hole collimator, according to validated protocol used at our institution [19]. Nodules were reported as cold, warm or hot.

### Sonographic Parameters and US-FNAC-

Real-time US was performed by a radiology team with experience in thyroid imaging at our institution. Internal components of the nodule were defined as solid, mixed, or cystic. US analyses for masses with mixed components were evaluated on the basis of internal solid components. The FNA was performed systematically in nodules ≥ 1 cm in diameter. US features defined size and suspicious parameters of malignancy [6,17] as following: hypoechogenicity, microcalcifications, border irregularity and central flow by Doppler study. The confirmed presence of one of these characteristics defined the nodule as positive at US findings (suspicious malignant nodule) being the FNA study indicated in nodule < 1 cm [6] diameter. If a nodule did not show suspicious features it was classified as probably benign (negative US findings). The nodule size and presence of other nodules within thyroid were also noted and classified as single/solitary vs. multinodular goiter. In multinodular goiter group the most suspicious nodule was included in the study. Longitudinal and transverse views of thyroid were obtained. The US-FNAC was performed in the nodules using a 22-gauge needle without local anesthesia. If the aspirate was hemorrhagic a 25-gauge needle was used. Aspiration was expressed on frosted-end glass slides, air-dried, and stained using Papanicolaou's method [20]. For each sample, at least three slides were obtained for cytological analysis.

### Cytological Features

**Figure 1:**
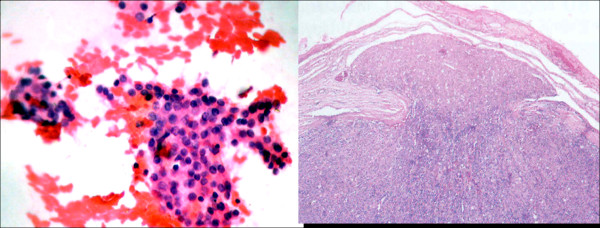
a) A Category IV of Bethesda System (follicle formation, high cellularity, microfollicles, scant colloid, and no nuclear features of papillary thyroid cancer - Papanicolaou's stain); b) Histology result confirmed minimal invasive follicular carcinoma.

Cytological analysis was based on the Bethesda classification system [21]. All slides from FNAC findings were re-analyzed by an expert cytopathologist of our Pathology Department (P.S.M) in order to confirm the results. After obtaining the previous pathology reports the cytologist reviewed them and reclassified the cases using the Bethesda system according to the microscopic features which were described on the existing pathology reports. This review of slides was blind to outcomes. The FNAC result was reclassified into six categories: unsatisfactory (I), benign (II), follicular lesion of undetermined significance (III), follicular neoplasm (IV), suspicious of malignancy (V) and malignancy (VI) (Table 3). Benign cytologic findings included colloid nodules, adenomatous hyperplasia, lymphocytic thyroiditis and toxic diffuse goiter. The indeterminate category (III and IV) included: follicular lesion, Hurtle Cell tumor, and an atypical presentation so that malignancy could not be excluded (Figure 1). Follicular neoplasm showed follicle formation, high cellularity, microfollicles, scant colloid, and no nuclear features of papillary thyroid cancer (PTC). The FNAC findings were considered suspicious for PTC when papillary structures were found, and also had nuclear enlargement, intranuclear inclusions or nuclear grooves. The unsatisfactory sample was define as the absence of at least six follicular cell groups, each one containing 10-15 cells derived from at least two aspirates of a nodule according to the American Thyroid Association (ATA) guidelines. We did not include the unsatisfactory samples in calculations. The findings of malignancy were confirmed by means of surgery.

**Table 3:** FNAC Result and Histology Correlation of Thyroid Nodules - Accuracy of FNAC in the Preoperative

FNAC	Final Histology		Total
Bethesda Category*	Benign	Malign	
II	24 (80%)	6 (20%)	30 (21.7%)
III	45 (93.8%)	3 (6.2%)	48 (34.8%)
IV	16 (47.1%)	18 (52.9%)	34 (24.6%)
V	05 (31.2%)	11 (68.8%)	16 (11.6%)
VI	-	10 (100%)	10 (7.3%)
Total	90	48	138 (100%)

The sensitivity, specificity, positive predictive value and negative predictive value of FNAC were 82.8%, 97.7%, 80%, 80.7%, respectively.

*FNAC: fine-needle aspiration cytology; II: benign; III: follicular lesion of **undeterminate **significance; IV: follicular neoplasm; V: suspicious of malignance; VI: malignant.

### Statistical Analysis

**Figure 2:**
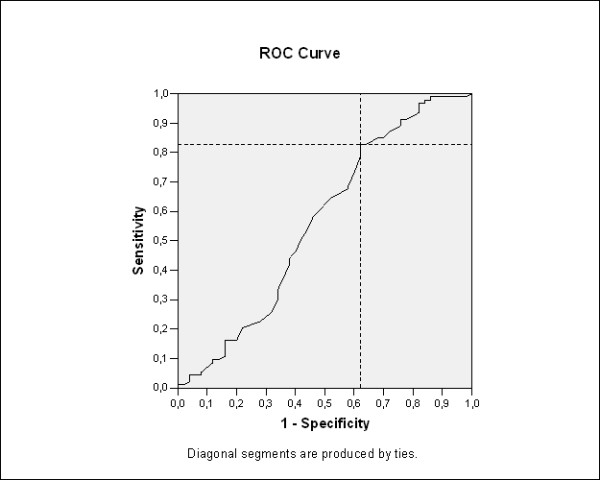
**Age cutoff of 38.5 years by ROC-curve analysis as an independent risk predictor factor of thyroid malignancy: sensitivity of 82.9% and specificity of 38%, with 56.1% of accuracy for malignancy in 143 patients at a single center**.

This study was approved by our institutional review board. For the univariate analysis, data was analyzed using chi-square test, or Fisher's exact test to categorical variables, and the non-parametric test (Mann-Whitney) to quantitative variables of the two groups (P < 0.05). For the multivariate analysis, a logistic regression model was applied to data, using the predictors of malignancy that was statistically significant in the univariate analysis. To analyze the relationship between age and thyroid nodule malignancy, we created a receiver operating characteristic (ROC) loop to identify cutoff points to enable identification of specificity and sensitivity of age related to thyroid cancer (Figure 2). Finally, we created a diagnostic predictor model based on data from the multivariate analysis and tested it for accuracy in prediction of thyroid malignancy. Statistical analyses were performed using the SPSS version 13.0.

## Results

**Figure 3:**
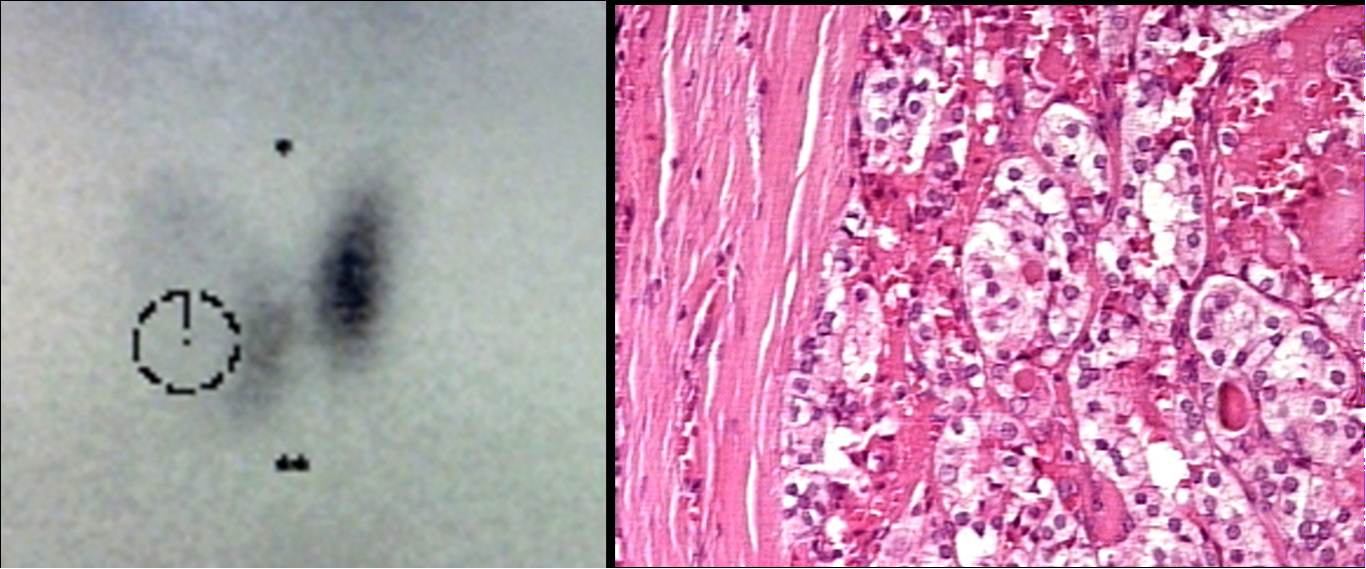
a) Scintigraphyc scan showing cold nodule in euthyroid female patient of 34 years old; b) Histology result confirmed benign follicular nodule (an adequately cellular specimen comprised of varying proportions of colloid and benign follicular cells arranged as macrofollicles and macrofollicle fragments).

Malignancy in thyroid nodules was associated to age and suspicious sonographic features. There were no differences in gender (AOR 1.35, 95% CI 0.53-3.42, P = 0.52), serum TSH levels (P = 0.11), FT4 levels, thyroid auto-antibodies (TAb), thyroid auto-immune disease and thyroid dysfunction (hypo and hyperthyroidism) between the two groups (Table 2). Patient's age was an independent clinical significant predictor of malignancy (AOR 2.95, 95% CI 1.34-6.46, P = 0.007) and an age cutoff of 38.5 years was applied to the ROC curve analysis (Figure 2). The multiple logistic regression to analyze gender, age, solitary nodularity, and TSH concentration confirmed a significant increased adjusted odds ratios (AOR) for malignancy in patients of > 39 years. The solitary nodules were not at increased risk (AOR 1.10, 95% CI 0.83-1.46, P = 0.48) in our study. Results of scintigraphyc showed the presence of cold nodules in 62.5% of malignant nodules (Figure 3) vs. cold nodules in 76.9% of benign (P = 0.33). The warm or hot nodules were not considered a predictive for malignancy on thyroid nodule investigation (P = 0.25).

The sonographic assessment showed a larger mean size in benign nodules (2.23 ± 1.82 vs. 2.87 ± 1.65 cm; P = 0.003) and a positive US in the malignant group, including: microcalcifications, central vascularity, border irregularity, and hypoechogenicity (Table 4). The nodule ≥ 2 cm in diameter was also a significant predictor of malignancy. After multivariate analysis, detection of simultaneously presence of age > 39 yrs., border irregularity, microcalcifications and nodule ≥ 2 cm in diameter by US study, a high accuracy to identify malignant thyroid nodules was shown (Table 5). The border irregularity and microcalcifications constituted the strongest predictors of malignancy after multivariate analysis. The hypoechogenicity and central flow were significant in the univariate analysis only.

**Table 4:** Ultrasound Parameters of Malignancy of the 143 Patients that underwent Thyroid Nodule Evaluation in a Single Center

Variables	Benign		Malign		*P-*value
	**N**	**%**	**N**	**%**	

Nodules (n)	93		50		
Nodule (number)/patient	1.58 ± 1.15		1.73 ± 1.31		0.51
Nodule size (cm)	2.87 ± 1.65		2.23 ± 1.82		0.003
Nodule size (range) [cm]	0.3 - 9.3		0.3 - 9.0		
Microcalcifications	12	14.1%	20	45.5%	< 0.001
Macrocalcifications	15	17.6%	04	9.09%	0.06
Border Irregularity	15	17.8%	34	75.5%	< 0.001
Hypoechogenicity	36	42.3%	32	71.1%	0.003
Hyperechogenicity	31	36.4%	06	13.3%	0.001
Size ≥ 2 cm	64	73.5%	23	46.0%	0.001
Internal Flow	13	21.6%	17	56.6%	< 0.001
Absent Flow	27	45.0%	06	20.0%	< 0.001

**Table 5:** Independent risk factors of thyroid malignancy from a single center: Predictor Model Accuracy of 81.7%**

Variables	Adjust Odds Ratio	95% Confidence Interval	*P-*value
Age at Diagnosis (≥ 39 years)	7.26	1.79 - 29.3	0.005
Microcalcifications*	10.28	1.62 - 64.8	0.013
Border Irregularity*	18.82	5.18 - 68.3	< 0.001
Size ≥ 2 cm*	6.20	1.74 - 22.1	0.005

*Ultrasound parameters

**Multiple logistic regression analysis considering age, nodule size and ultrasound features at presentation

## Discussion

The predictor model shows a high accuracy (> 80%) for malignant thyroid nodule when it includes age (≥ 38.5 years.), border irregularity, microcalcifications, and nodule size (≥ 2 cm) using high-resolution ultrasound. In our study, scintigraphyc study was not useful to differentiate the two groups. The number of nodules (solitary vs. multiple) did not predict malignancy. No other clinical or laboratory parameters were significant in this study, including auto-immune disease and TSH level. Therefore, a risk stratification scheme would theoretically help both the patient and the surgeon to make a better decision upon the extent of recommended surgery.

Age in thyroid nodule patients was identified as an independent predictor for malignancy with an age cut off of ≥ 38.5. Baier et al (2009) reviewed reports of 944 thyroid nodules of four sonographic features and found statistical significance in malignant nodules in young patients (≤ 45 years) and solid nodule morphology [22]. Several studies have tried to predict malignancy in thyroid nodules with indeterminate or suspicious FNA findings according to the M.D. Anderson Cancer Center series [23]. The ROC-curve of our data shows a different age cutoff to predict malignancy during thyroid nodule evaluation, providing good accuracy and high sensitivity rate. In fact, the application of the International Union against Cancer (AJCC/UICC) classification system based on pTNM parameters and in age is recommended for tumors of all types, including thyroid cancer [6], because it provides a useful shorthand method to describe the extent of the tumor. Age is one of the criteria, with cutoff of over 45 years that is in disagreement with the findings showed in our data, which agree with the Banks and Baier et al study [16,20].

The importance of TSH levels as a predictor of malignancy in thyroid nodule evaluation, have been discussed in recent studies showing that an elevated serum TSH concentration might be associated to increased risk of different thyroid cancers in patients with nodular goiter [11,12,15]. Higher TSH values, even within normal ranges, have been associated with a greater risk of thyroid malignancy in some studies [11-15]. Boelaert et al (2006) studied 1.500 consecutive patients without overt thyroid dysfunction and found a significant increase in adjusted odds ratios (AORs) for the diagnosis of malignancy in subjects with serum TSH 1.0-1.7 mU/liter compared to TSH less than 0.4 mU/liter (AOR 2.72), with further increases being evident in those with TSH 1.8-5.5 mU/liter (AOR 3.88). Males, younger patients, and those with clinically solitary nodules were also at increased risk [12]. We did not observed correlation of gender or solitary nodule in our data. The TSH concentration was not significant after multivariate analysis in our study in accordance with some authors [15] and in disagreement with other ones [11,12], remaining in this way unclear to date and needing further investigation.

In our data correlation with thyroid auto-immunity and malignancy was not found. In the majority of the previous retrospective studies, there is a support for the correlation between thyroid malignancy and Hashimoto's thyroiditis (HT) [24-30]. A current review of the American Thyroid Association guidelines for thyroid nodules and thyroid cancer stated that rate of malignancy in nodules in thyroid glands involved with HT could be possibly higher [6,31]. In a recent study by Anil et al (2010), a malignancy rate of 1.0% in HT group (2 out of 191 nodules) vs. 2.7% in the control group (19/713) was demonstrated, although no statistical significance was found even at higher TSH levels [31] that is similar to our results.

Thyroid ultrasound is used to evaluate index of nodule size, location, characteristics, number and presence of additional thyroid nodules and to detect suspicious appearance of lymph nodes [17]. Nodule size has been pointed out not to be a predictive of malignancy [6,11,12,18]. Patients with multiple thyroid nodules have the same risk for malignancy as those with solitary nodules. Is recommended that all patients with nodular thyroid glands should be submitted to US evaluation [6,18]. Our data showed correlations of thyroid malignancy with nodules which presented microcalcifications, border irregularity, size ≥ 2 cm, central flow by Doppler and hypoechogenicity after US study. After a multiple logistic regression border irregularity and microcalcifications were the strongest predictors of malignancy in thyroid nodule, followed by the nodule over 2 cm in diameter.

Gonzalez-Gonzales (2010) evaluated the efficiency of diagnostic of sonographic findings and compared to those of FNA biopsy of thyroid nodules to study US characteristics of 341 thyroid nodules. The multivariate logistic regression revealed that the only variable, which kept a significant association with malignancy, was the presence of microcalcifications [18]. These data confirm the study by Li QS (2010) who retrospectively reviewed 115 nodules (104 patients) with PTC. They also analyzed thyroid nodules and cervical lymph nodes size, border, calcification, echotexture, hemodynamic on US. The microcalcifications showed an increased in suspicion for malignancy of thyroid nodule [32]. A hypoechoic thyroid nodule with increased internal vascularity, ill-defined border and microcalcifications, PTC was strongly suggested, which is similar to our data.

The color Doppler analysis was not correlated to thyroid malignancy in our study, which agrees with currently data in the literature. Moon et al (2010) evaluated 1083 thyroid nodules, 814 benign and 269 malignant. The central flow was frequently seen in benign nodules and the absence of vascularity was more frequent in malignant nodules. Vascularity itself or a combination of vascularity and gray-scale US features was not as useful as the use of suspicious gray-scale US features alone for predicting thyroid malignancy [13], similar to the data of Cantisani et al (2010) of 1.090 assessed patients [14]. In their study, they concluded that pattern III cannot be used to predict malignancy with confidence, and FNA still is mandatory to remove the nature of the nodule. Choi et al (2009) followed up 165 patients with indeterminate cytology diagnosed as follicular neoplasm and no difference in malignancy incidence on gender; age (≥ 45 years), nodule size and US features were found. Only central color Doppler flow was predictive for malignancy in follicular neoplasm [33]. However, Anil et al (2010) showed that US features of nodule echogenicity, structure, margin, and Doppler flow were similar in patients with Hashimoto's thyroiditis and in control group [31].

Banks et al (2008) proposed a risk score analysis based on patient age (50 yrs), nodule size (2.5 cm) and cytopathological features (atypia) for patients with indeterminate or suspicious FNAC [16]. They observed a nonlinear relationship between age and risk of malignancy, and patients at both age extremes were more likely to have malignant thyroid nodules.

A predictor model was created using variable of age (> 39 years), border irregularity, microcalcifications and nodule diameter (>2 cm) to identify thyroid malignancy with good accuracy (>80%). Is important to highlight that to understand the combination of age and US parameters in malignancy prediction is essential for clinicians to make decisions, and to guide surgical definition in many cases. The TSH level, the presence of auto-immune disease or scintigraphyc study was not useful to make differentiation in the two groups. Male gender, solitary nodule or Hashimoto's thyroiditis were also not considered predictors of malignancy in our study.

Risk prediction, based on clinical and US parameters, should be used as an adjunct the findings of FNA aiming to identify patients who require further investigation and/or surgical intervention. Prospective studies are required to define the role of this risk prediction to improve clinical management in a larger patient population.

## Competing interests

The authors declare that they have no competing interests.

## Authors' contributions

FFRM carried out the cytopathological review, ultrasound and data based collected, participated in its design and statistical analysis. PSM participated in the cytopathology analysis and study design. BPS and ATP carried out the initial data based collected. EJP participated in the design of the study and performed the statistical analysis. JV and DWZW conceived of the study, and participated in its design and coordination. All authors read and approved the final version of the manuscript.
